# Delay in cutaneous melanoma diagnosis: Sequence analyses from suspicion to diagnosis in 211 patients

**DOI:** 10.1097/MD.0000000000004396

**Published:** 2016-08-07

**Authors:** Marcus H.S.B. Xavier, Ana P. Drummond-Lage, Cyntia Baeta, Lorena Rocha, Alessandra M. Almeida, Alberto J.A. Wainstein

**Affiliations:** a Faculty of Medical Sciences, Belo Horizonte, MG, Brazil; b Oncad, Surgical Oncology, Belo Horizonte, Brazil.

**Keywords:** delay, diagnosis, melanoma

## Abstract

Advanced melanoma is an incurable disease with complex and expensive treatments. The best approach to prevent melanoma at advanced stages is an early diagnosis. A knowledge of factors associated with the process of detecting cutaneous melanomas and the reasons for delays in diagnosis is essential for the improvement of the secondary prevention of the disease.

Identify sociodemographic, individual, and medical aspects related to cutaneous melanoma diagnosis delay.

Interviews evaluated the knowledge of melanoma, signals, symptoms, persons who were suspected, delays in seeking medical attention, physician's deferrals, and related factors of 211 patients.

Melanomas were self-discovered in 41.7% of the patients; healthcare providers detected 29.9% of patients and others detected 27%. The main component in delay was patient-related. Only 31.3% of the patients knew that melanoma was a serious skin cancer, and most thought that the pigmented lesion was not important, causing a delay in seeking medical assistance. Patients (36.4%) reported a wait interval of more than 6 months from the onset of an observed change in a pigmented lesion to the first visit to a physician. The delay interval from the first physician visit to a histopathological diagnosis was shorter (<1 month) in 55.5% of patients. Improper treatments without a histopathological confirmation occurred in 14.7% of patients. A professional delay was related to both inappropriate treatments performed without histopathological confirmation (*P* = 0.003) and long requirements for medical referrals (*P* < 0.001).

A deficient knowledge in the population regarding melanoma and physicians’ misdiagnoses regarding suspicious lesions contributed to delays in diagnosis.

## 1 Introduction

Skin cancer is the most common cancer in Brazil and represents 25% of all registered malignancies globally.^[1]^ Cutaneous melanoma accounts for only 4% of skin cancers and despite being less frequent, is the most serious cancer due to its specific early competence to establish metastases, cost of treatment and number of lives lost. Globally, incidence has increased over the last 30 years,^[2,3]^ and cases of death by melanoma has increased in some countries,^[4]^ while stabilizing and declining in others.^[5,6]^ Despite the therapeutic advances in treating advanced melanoma over the past 5 years,^[7]^ the actions of primary and secondary prevention are still decisive for mortality reduction,^[8]^ as demonstrated in Australia. Prevention campaigns and early diagnosis have been effective^[9]^ as the main strategy for mortality reduction based on melanoma identification and early treatment of the subject with a suspected lesion.^[10]^

Despite the importance of early detection in preventing mortality from melanoma, little is known regarding how patients with the disease reach diagnosis.^[11]^ Risk of death from melanoma is related directly to the Breslow thickness of the primary lesion,^[12,13]^ and there is a positive correlation between tumor thickness and the delay to identify a lesion as suspicious.^[14–16]^ Therefore, a reduction in the diagnostic delay, considering both the recognition and the search for assistance by the patient, as well as the diagnosis and proper assessment by the doctor, are critical in predicting the outcome. Currently, the regulatory authorities in most countries express dissatisfaction regarding the high cost of the new but very effective treatments for advanced melanoma. It is difficult to deny this access, benefit and hope to these populations. Rather than spending time discussing the cost associated with the handling of metastatic disease, the most effective approach is to invest in prevention and early diagnosis. To achieve this, understanding the process from melanoma diagnosis to treatment is essential for success.

## 2 Patients and methods

A longitudinal cohort study was performed through a retrospective review of patients referred to Oncad—Surgical Oncology with a histological confirmation of cutaneous melanoma. A prospective data collection based on a questionnaire constructed for the research groups was performed from November 2014 to April 2015. Data included the location of the lesion and the histological data set for the melanoma.

A detailed questionnaire was constructed, which included sociodemographic variables, knowledge about melanoma, time interval to diagnosis, time delay, and related factors. There was also a query about the person who first recognized the melanoma. When patients self-detected their lesions, the signs and symptoms were registered. We investigated the length of patient-related delays in seeking medical attention, reasons for these delays, misdiagnosis situations, inadvertent procedures, and time until definitive treatment after the first medical examination.

Self-detection was assumed when patients themselves first noticed a change in a pre-existing pigmented lesion or became aware of a new pigmented lesion later confirmed as melanoma. Patients were asked about the time interval between the initial suspicion and first professional consultation (patient delay) and between the first professional consultation and surgical treatment with a histopathological diagnosis (professional delay). Melanoma knowledge was assessed by considering the patient's melanoma information prior to the diagnosis. Patients who declared melanoma “a serious skin cancer” or “pigmented skin cancer” were defined as patients with melanoma knowledge.

Written informed consent was obtained from each patient. Most patients were interviewed within 1 year of diagnosis, and patients with imprecise or partial answers were censored. All patients were aware that they had melanoma at the time of the interview. Data protection was respected, as the patients were anonymized.

Clinical data, including the site and histopathological subtype of the melanoma, were defined by the histopathological reports. The tumor site was classified as head/neck, anterior trunk, back, upper extremities, lower extremities, or palmar/plantar aspects. Melanoma subtype was assessed according to Clark and staged according to Breslow. The skin type of each patient was assessed by visual inspection according to the Fitzpatrick Classification.^[17]^ The study was approved by the institutional review board under No. 846.018.

### 2.1 Statistical analysis

Data analysis was performed using software (SPSS, version 17.0). Descriptive statistics were generated to characterize the study population. The Student *t* test was used to compare the means. The chi-squared or Fisher exact test was used to compare proportions, and the Mann–Whitney *U* test was used to test the equality of medians between samples. A *P* value of 5% was considered to be significant.

## 3 Results

In total, 221 patients were eligible for this study. Of these, 4 patients refused participation, and 6 were censored because of imprecise information, leaving 211 patients for inclusion. Out of these patients, 103 were men (48.8%) and 108 were women (51.2%). The mean age was 53.3 years (range 19–96 years). Most patients were married (77.4%), had skin type I or II (79.3%), and had a university degree (62.1%). The most frequent histologic subtype was superficial spreading melanoma (63.5%), followed by nodular melanoma (12.4%) (Table 1). The back was the most common site (24.6%), followed by the lower extremities (23.2%). Histopathological examination revealed that 61.6% of our patients had a tumor thickness ≤1 mm (Table 1) with a median Breslow value of 1.96 mm when excluding in situ patients.

**Table 1:**
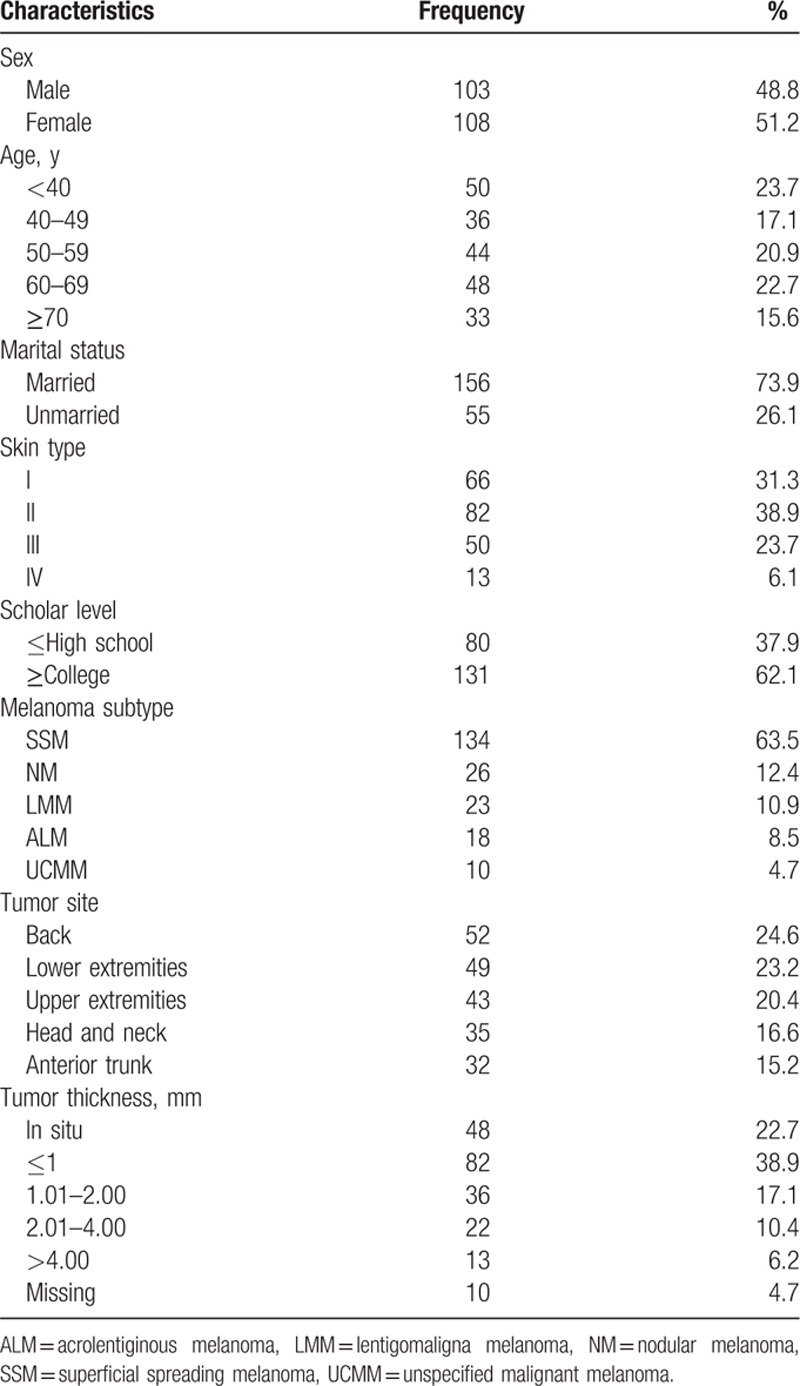
Characteristics of the study population (n = 211).

The majority of patients (41.7%) reported that they were the first to recognize the melanoma. Others who first identified the tumor were family members and friends (14.7%), spouses (12.3%), health professionals (29.9%), and others (e.g., hairdressers) (1.4%). Most of health professionals who recognized the melanoma were dermatologists (19%), other physicians (9.5%), and other health professionals (1.4%) (e.g., physiotherapist). Although self-detection was more common in females (64.8% vs 35.2%), physician detection was more common in males (58.5% vs 41.5%; *P* = 0.001).

By analyzing the first person who suspected the lesion, we observed that patients with melanoma discovered as incidental findings showed thinner tumor thickness than those tumors initially detected by the patients themselves (Table 2).

**Table 2:**
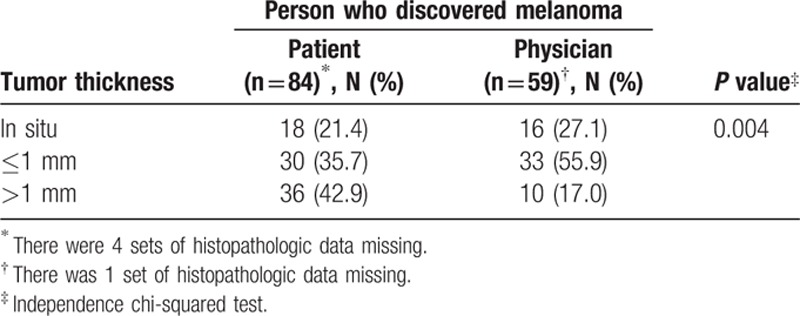
Relation between tumor thickness and the person who discovered the melanoma.

The symptoms most noticed were an increase in diameter (45.5%), an elevation (31.8%), change in shape (19.3%), ulceration or bleeding (19.3%), and color change in the primary lesion (18.2%). Some patients (16%) were also aware of an altered sensation (itch) related to the lesion. Some patients reported more than 1 feature when asked about the symptoms of the lesion. There was no difference in patient delay between those who experienced changes in size, shape, or color in a pigmented lesion and those who presented bleeding, ulceration, or elevation lesions (*P* ≥ 0.95).

To determine the effect of anatomic site (i.e., lesion visibility) on the pattern of detection of melanoma, we stratified the anatomic regions into 2 categories: “exposed” and “occult.” Anatomic regions were considered “exposed” (face, ear, forearm, hand, dorsal foot, anterior neck, arm, thigh, leg, and trunk) or “occult” (scalp, sole of the foot, posterior neck, arm, thigh, leg, and trunk). It was observed that 39% of patients had melanoma arising on a visually occult primary site and 61% from an exposed area, without gender influence. In contrast, self-detected melanoma was more frequent among individuals with “exposed” lesions compared with those with “occult” lesions (50.4% vs 28%, respectively) (*P* = 0.002).

When asked about their knowledge of cutaneous melanoma, only 31.3% patients reported understanding the real meaning of “melanoma.” Men were slightly more aware of the existence of cutaneous melanoma than women (33% vs 29.6%) (*P* = 0.524). More than 80.3% of the patients with knowledge of the meaning of melanoma had a high educational status (at least college) (*P* < 0.001). Auto detection was more frequent among patients with Fitzpatrick III compared with patients with Fitzpatrick I or II (55.6% vs 35.8%, respectively) (*P* = 0.012). There was no significant association between patient's phenotype (considering just Fitzpatrick grade, as nevus count was not available), melanoma knowledge, doctor detection, and patient delay. There was no significant association between family history and melanoma knowledge.

Excluding incidental diagnosis by physicians, the mean delay between noticing the appearance of a new lesion or the onset of changes and the first medical consultation was 5 months. Medical attention occurred within 1 month in 23.2% of patients. Among those who waited before seeking medical attention, 23.8% delayed for up to 3 months, 16.6% for up to 6 months, 11.1% for up to 12 months, and 25.2% waited more than 12 months.

The main reason for delay was an impression that the lesion was benign (43.2%) followed by a refusal in looking for medical assistance (38.5%). Interestingly, 9.5% of the patients mentioned that they were too busy to consult a physician and could not detect their lesions themselves because of its anatomical site (2%) or were afraid of the physician's diagnosis (1.4%). Other reasons reported were medical misinformation that the lesion was benign and a difficulty to consult with a specialist (Table 3).

**Table 3:**
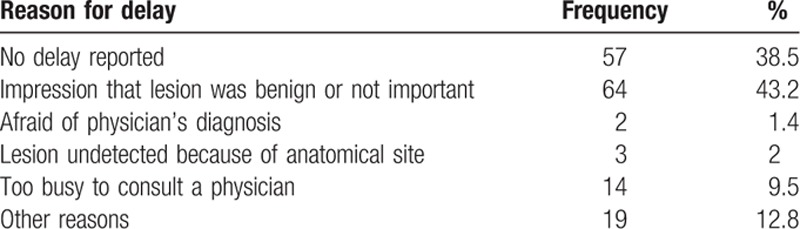
Reasons for patient's delay in seeking medical advice (excluding medical incidental diagnosis; N = 148).

Initial treatment without histopathological confirmation (e.g., ointments, electrocautery, liquid nitrogen, curettage) occurred in 14.7% (31/211) of our patients. Both general physicians and dermatologists performed these treatments. Approximately half of these patients (14/31) were diagnosed 1 year after their first medical consultation.

There was a significant association between diagnosis delay and previous management without a histopathological confirmation, which postponed effective diagnosis and treatment. There was a higher proportion of patients who underwent previous inappropriate treatment among those who had a definitive diagnosis within less than a month (7.7%) (*P* = 0.003). The association was also significant between the diagnosis and the number of physician visits (*P* < 0.001). There was a higher proportion of individuals who sought assistance at least twice among those who had delayed diagnosis for a month or more (59.6%) than among those who had a definitive diagnosis in <1 month (27.4%) (Table 4).

**Table 4:**
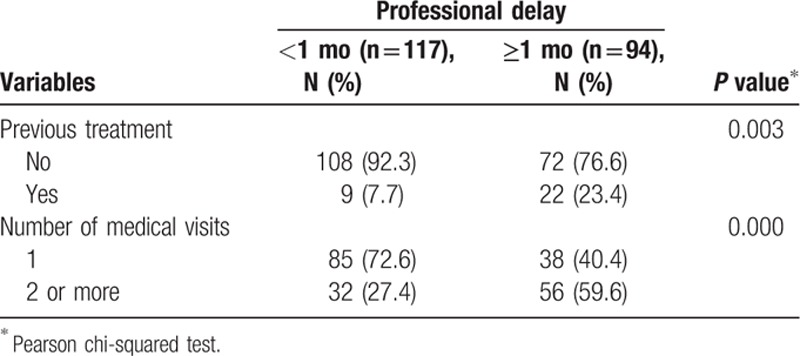
Professional delay in excision of a cutaneous melanoma and the assessed variables.

## 4 Discussion

Cutaneous melanoma is visible and potentially detectable in its initial phases, and many different individuals can be involved in its identification. Early diagnosis is closely related to a better chance of cure, survival, and lower treatment costs, leading to a major health efficacy. Nevertheless, many patients present with invasive disease and a long history of melanoma signs and symptoms before seeking medical attention. The reasons for a diagnostic delay in patients with signs and symptoms of melanoma are multifactorial and include a lack of knowledge of melanoma among the public and medical professionals, as well as a failure of patients and doctors to examine the skin routinely. Few studies have investigated delays in diagnosis of cutaneous melanoma and the related reasons.^[15,16,18–26]^

In this study, the majority of patients (41.7%) reported that they were the first to recognize their melanoma. Other people who were first alerted to the skin tumor were family members/friends (14.7%) and spouses (12.3%). According to literature, patients with skin type I or II have a higher risk of melanoma development. Frequently these patients have many typical and atypical nevus what turn more difficult a melanoma detection among these pigmented lesions. Also this population usually has more dermatological appointments and attributed the detection responsibility to its physician. In opposite, some patients with Fitzpatrick III or more develop melanoma as a unique lesion with typical “ugly duck signal” what turns out easier to get patient attention on it.^[17]^

In the literature, approximately half of melanomas are discovered by the person with the lesion.^[16,18,23,25,27–30]^ Melanoma was suspected by the spouse, another family member, or a friend in one-fourth of the patients. For the remaining one-fourth of the patients, the lesion was discovered by a medical provider upon physical examination (accidental discovery).^[16,19–21,27–29]^ This information strengthens the importance of many participants committed to the diagnosis of melanoma and the need for proper training, as well as reinforcing the importance of full skin examination for secondary prevention. Consistent with other studies, we noticed that melanomas detected casually in routine clinical examinations have the characteristic of being thinner than those detected by the patients themselves, suggesting that awareness of the disease by physicians and the lay public is critical for early detection.^[11,22–24,29]^

It has been reported that an occult primary site is more common in back lesions in men and lower limb lesions in women.^[11,27]^ Regardless of the anatomical region, it has been shown that women self-detected more lesions than men, and men rarely found the lesions on their back.^[15,27]^ In this study, self-detection was more common in females (64.8% vs 35.2%) (*P* < 0.001) and detection by spouses occurred in 12.3% of the patients. In the literature, it has been shown that women also detect more lesions on their spouses.^[11,16,19,27]^ Women are more likely to perform skin self-examination^[11,31]^ and are more knowledgeable about the disease,^[11,27,31]^ although the latter has not been observed in this study.

The reasons for delays in diagnosis in patients with signs and symptoms of melanoma are multifactorial, and include a lack of knowledge of the disease among the population, as well as a failure of patients and physicians to examine the skin routinely.^[11]^ In our study, the leading component of delay was related to the patient.^[9,15,20]^ Although there is some variation in other studies, our average delay of 5 months was comparable to previously reported patient-related delays of approximately 2 to 9.8 months^[16,19,20,23,25]^ (Table 5). While there was an average delay of 2 months reported in the study by Richard et al,^[19]^ the author states that this interval is very long for this population, as many campaigns in France were conducted at this time. We believe that our delay in Brazil of 5 months could be decreased if we had a national melanoma awareness campaign similar to other countries.

**Table 5:**
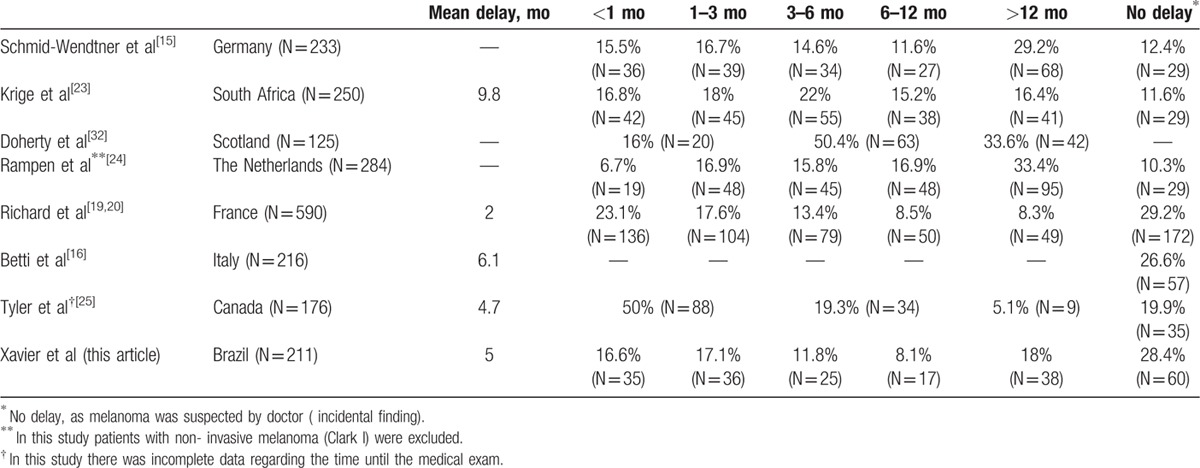
Patient delay: interval in months between the patient's initial observation and the first professional consultation.

We observed that only 31.3% of patients reported having previous knowledge about melanoma. This percentage is lower than in other countries: France (53%),^[19,20]^ Italy (60%),^[16]^ Germany (82.3% and 85.3%),^[15,28]^ and Canada (93%).^[25]^ As reported in the literature, we observed an association between the educational level and better knowledge of melanoma.^[16,18,21,22,24,31]^ Furthermore, it is known that the highest educational level is associated with higher rates of melanoma self-detection.^[19,20,27]^ This information is important, as the patients of this study represented an upper group in the Brazilian population with a high income, a high level of education, and unrestricted access to a private health system. It is known that 79.5% of Brazilians have not completed elementary school^[33]^; however, 62.1% of the study participants had completed the college level. This is relevant, as only approximately one-fourth of the Brazilian population uses a private health system,^[34]^ which allows for better access to medical specialists that theoretically would reduce the delay in diagnosis and treatment despite the Brazilian public system, where some medical access is limited or not accessible.

Unexpectedly, we noticed failures in the identification of melanoma in individuals who could recognize the early signs (change in size, shape, and color) but did not seek medical care in less time when compared with those who recognized the late signs (ulceration, bleeding, and elevation). This fact harms the quality and the benefit of the knowledge and awareness about melanoma in this population. The presence of a bleeding tumor (i.e., a sign of aggressive disease) was described by 19.3% of respondents, similar to previous reports.^[22–24,35]^

Cassileth et al^[22]^ have shown that patients who experienced changes in size, color, or elevation in a pigmented lesion waited an average of 1 year before referring to a physician for evaluation. Symptoms of bleeding or ulceration resulted in a shorter delay in presentation, but these symptoms were associated with deeper lesions.

Richard et al^[19]^ observed that even though many patients are able to detect their tumor earlier, some patients did not take advantage of this situation, as the initial signs were not always interpreted as those who required a prompt visit to a doctor. Indeed, patients who noticed discreet changes in the colors form or size (asymmetry, border, colors, and dermoscopic rule) tended to seek medical attention later than others. We did not observe these results in our study, as we did not see a difference in the time to diagnosis from patients with signs of early and advanced melanoma.

Oliveria et al^[36]^ investigated the relationships between patient knowledge, awareness, and delay in seeking medical attention for melanoma. The study population was comprised of 255 patients and showed that 63.1% of subjects reported bleeding and 65.9% reported a “scab that won’t heal” as melanoma finding indicators. Moreover, early signs of melanomas have been reported in smaller proportions: color change (46.7%), increase in diameter (28.2%), and change in form (40.8%). These data indicate that patients failed to recognize the early signs and symptoms of melanoma as we confirmed, likely due to the lack of information that it is a dangerous cancer.

A few studies have evaluated the role of physicians in the diagnosis delay of cutaneous melanoma.^[20–25]^ The prognosis of patients with melanoma is usually not influenced by the doctor,^[20,22,23,26]^ although some professionals still have inappropriate attitudes toward melanoma being harmful. Delaying diagnosis and performing improper treatments while providing inadequate information and inappropriate approaches contribute significantly to an impaired patient's early diagnosis.^[20]^ This fact becomes more severe in patients with melanoma with atypical presentations or in unusual locations (sole and nail apparatus), further contributing to the delay in diagnosis.^[26]^

The mean time between the first medical consultation and the final diagnosis in this study (4.1 months) was greater than those found in the literature: 1.3 months in South Africa,^[23]^ 1.5 months in Italy,^[16]^ 2 months in Germany,^[15]^ 3 months in United States,^[22]^ and 3.9 months in Canada.^[25]^ We observed that 55.5% of patients were diagnosed within the 1st month after consultation. This proportion is lower than that observed in studies conducted in other countries: 87.6% in South Africa,^[23]^ 85.8% in France,^[19,20]^ and 74.7% in Germany.^[15]^ We observed that excessive medical referral could delay the early diagnosis of patients, possibly because of the inexperience or insecurity of physicians in making a melanoma diagnosis. Thus, we believe that the long delay in melanoma diagnosis in this high-income Brazilian population should be even longer in those patients who depend on the Brazilian National Health System, which is an inefficient public health system, where the wait time for a consultation with dermatologists and surgeons is very long. Future studies will include an extrapolation of this work, repeating this study in a low-income population and comparing how socioeconomic status may affect melanoma diagnosis, stage, and prognosis.

## 5 Conclusion

Although obtaining data for this study depended on patients’ memories, and therefore a recall bias could not be excluded, we tried to minimize the selection bias by the consecutive recruitment of patients seen at our unit and the conducting of all interviews under the supervision of researchers.

The patients themselves and those who close to them are primarily responsible for discovering melanoma but also for the delay in seeking medical care. Despite the high socioeconomic level of the participants, there was a significant unawareness about melanoma and the recognition of its early signs.

It was observed that the gap between medical consultation and a definitive diagnosis was higher than in some studies performed in other countries. Another concern is the evidence that many doctors are not able to identify early melanoma, which would contribute to the delay in diagnosis and consequently lead to the treatment of advanced disease with a poor prognosis and a high cost of therapies.

Professional programs should focus on health professionals other than only dermatologists. These programs should emphasize the importance of skin inspection for the detection of suspicious lesions, as well as establish and teach health professionals and lay people which lesions are harmful.

We have documented the process of melanoma suspicion and diagnosis in Brazilian patients who have the highest socioeconomic status and access to all of the necessary facilities to receive professional and medical attention. Our findings were not satisfactory, and we noted that further studies are required to reinforce the need to improve the level of knowledge about melanoma in our society and among health professionals to include population awareness and early melanoma diagnosis as a health public need.

## Acknowledgments

The authors thank Dr Isabel Gomes for assistance with statistical analysis, patients with melanoma for strategic contributions, and our colleagues and professors from the Postgraduation Program at Medical Science Minas Gerais School of Medicine for comments that greatly improved the manuscript.
